# A hybrid classical-quantum approach to speed-up Q-learning

**DOI:** 10.1038/s41598-023-30990-5

**Published:** 2023-03-08

**Authors:** A. Sannia, A. Giordano, N. Lo Gullo, C. Mastroianni, F. Plastina

**Affiliations:** 1 Dipartimento di Fisica, Università della Calabria, 87036 Arcavacata di Rende, (CS) Italy; 2 Institute for Cross-Disciplinary Physics and Complex Systems (IFISC) UIB-CSIC, Campus Universitat Illes Balears, 07122 Palma de Mallorca, Spain; 3 ICAR-CNR, 87036 Rende, Italy; 4 INFN, gruppo collegato di Cosenza, Cosenza, Italy; 5 Quantum Algorithms and Software, VTT Technical Research Centre of Finland Ltd, Espoo, Finland

**Keywords:** Information theory and computation, Quantum information

## Abstract

We introduce a classical-quantum hybrid approach to computation, allowing for a quadratic performance improvement in the decision process of a learning agent. Using the paradigm of quantum accelerators, we introduce a routine that runs on a quantum computer, which allows for the encoding of probability distributions. This quantum routine is then employed, in a reinforcement learning set-up, to encode the distributions that drive action choices. Our routine is well-suited in the case of a large, although finite, number of actions and can be employed in any scenario where a probability distribution with a large support is needed. We describe the routine and assess its performance in terms of computational complexity, needed quantum resource, and accuracy. Finally, we design an algorithm showing how to exploit it in the context of Q-learning.

## Introduction

Quantum algorithms can produce statistical patterns which are hard to manipulate on a classical computer; in turns, they may, perhaps, help recognize patterns that are difficult to identify classically. To pursue this basic idea, a huge research effort is being put forward to speed up machine learning routines by exploiting unique quantum properties, such as superposition, coherence and entanglement^1, 2^.

Within the realm of machine learning, the Reinforcement Learning (RL) paradigm has gained attention in the last two decades^3, 4^, as, in a wide range of application scenarios, it allows modeling an agent that is able to learn and improve its behavior through rewards and penalties received from a not fully known environment. The agent, typically, chooses the action to perform by sampling a probability distribution that mirrors the expected returns associated to each of the actions performed, conditioned to the state of the environment. The aim of the RL procedure is to maximize the total reward that corresponds to the achievement of a given task. This is obtained by devising a stochastic strategy to train the agent in performing a series of actions, each picked from a given set, which maximizes the total reward. The final output of the RL is a conditional probability distribution that correlates the state of the environment with the action to be taken by the agent to modify its state.

It turns out that the RL performances can be improved by the use of quantum routines, as recently reviewed in^5^. To date, various promising proposals have been put forward that exploit quantum accelerators to speed-up RL, including, e.g., the speed-ups of projective simulations^6–8^, quantum models for RL policies with quantum circuits^9^ and Boltzman machines^10, 11^, applications in measurement-based adaptation protocols^12, 13^, and their implementations in photonic platforms^14, 15^. Moreover, in the same context, it is worth mentioning that some strategies based on the Grover’s algorithm^16^ have been proposed to generate the action probability distribution of a learning agent^17, 18^, which are suitable when the number of actions is finite.

Along this line, we introduce a novel algorithm that differentiates from the other Grover’s algorithm-based approaches mainly for our exploitation and coordination of the actions of multiple oracles that are associated with action subsets. As we will show below, this allows us to tune the probability distributions of the subsets in the exploration and exploitation phases. Moreover, our method generalizes those mentioned before as it is able to approximate, in principle, any desired probability distribution, thus overcoming the existing limitations in applying the standard Grover’s algorithm. Furthermore, our procedure does not require a prior knowledge of the probabilities to assign, as assumed in previous works^19, 20^. Following the classification proposed in^21^, our algorithm falls into the CQ framework, with a classical generating system and a quantum data processing device.

Specifically, in this work, we first introduce a routine to encode and update a probability distribution onto a quantum register and then we show how to embody it into a Q-learning based RL algorithm. The actions are clustered in a predetermined number of subsets (classes), each associated to a range with a minimum and a maximum value of the expected reward. The cardinality of each class is evaluated in due course, through a procedure built upon well-known quantum routines, i.e., quantum oracle and quantum counting^22, 23^. Once this information is obtained, a classical procedure is run to assign a probability to each subset, in accordance to any desired distribution, while the elements within the same class are taken to be equally likely. This allows one to tune probabilities, in order to, e. g., assign a larger chance to the actions included in the range with maximum value of the expected reward. The probability distribution can also be changed dynamically, in order to enforce exploration at a first stage (allowing some actions associated to a low probability to be chosen) and exploitation at a second stage (to restrict the search to actions having a higher likelihood of occurrence). The quantum routine presented here allows re-evaluating the values after examining all the actions that are admissible in a given state in a single parallel step, which is possible due to quantum superposition.

Besides the RL scenario, for which our approach is explicitly tailored, the main advantageous features of the routine could be also exploited in other contexts where one needs to sample from a probability distribution, ranging from swarm intelligence algorithms (such as Particle Swarm Optimization and Ant Colony Optimization^24^), to Cloud architectures (where the objective is to find an efficient assignment of virtual machines to physical servers, a problem that is known to be NP-hard^25^). After presenting the quantum routine in detail in section “Preparing a quantum probability distribution”, we focus on its use in the RL setting in section “Improving reinforcement learning”, to show that it is, in fact, tailored for the needs of RL with a large number of state-action pairs. In section “Additional features of the algorithm” we give an assessment of the advantages of our quantum accelerated RL over a pure classical algorithm and of the needed quantum resources. Then, a more technical section follows, discussing details of the probability encoding routine, and evaluating its complexity and precision (section “Details on the probability distribution encoding algorithm”). Finally, we draw some concluding remarks in section “Conclusions”.

## Preparing a quantum probability distribution

Here, we introduce the quantum routine which encodes a classical probability distribution into a Quantum Register (QR). Let us assume that we have a random variable whose discrete domain includes *J* different values, $\{x_j:j=1, \ldots , J\}$, which we map into the basis states of a *J*-dimensional Hilbert-space. Our goal is to prepare a quantum state for which the measurement probabilities in this basis reproduce the random variable probability distribution: $\{p_{x_1},p_{x_2},\cdots ,p_{x_{J}}\}$.

The quantum routine starts by initializing the QR as:

1 $$\begin{aligned} \left| {\psi }\right\rangle =\frac{1}{\sqrt{J}} \sum _{k=1}^{J}\left| {x_k}\right\rangle \left| {1}\right\rangle _a \equiv \left| {\phi }\right\rangle \left| {1}\right\rangle _a\ \end{aligned}$$

where $\left| {\phi }\right\rangle$ is the homogeneous superposition of the basis states $\{\left| {x_k}\right\rangle \}$, while an ancillary qubit is set to the state $\left| {1}\right\rangle _a$. In our approach, the final state is prepared by encoding the probabilities related to each state sequentially, which will require $J-1$ steps.

At the *i*-th step of the algorithm ($1\le i < J$), Grover’s iterations^16^ are used to set the amplitude of the $\left| {x_i}\right\rangle$ basis state to $a_i=\sqrt{p_{x_i}}$. In particular, we apply a conditional Grover’s operator: ${\mathbb {I}}\otimes \hat{\varvec{\Pi }}_{a}^{(0)} + \hat{\varvec{G}}_i\otimes \hat{\varvec{\Pi }}_{a}^{(1)}$, where $\hat{\varvec{G}}_i=\hat{\varvec{R}}\hat{\varvec{O}}_i$ and $\hat{\varvec{\Pi }}_{a}^{(y)}$ is the projector onto the state $\left| {y}\right\rangle _a$ of the ancilla ($y=0,1$). It forces the Grover unitary, $\hat{\varvec{G}}_i$, to act only on the component of the QR state tied to the (’unticked’) state $\left| {1}\right\rangle _a$ of the ancillary qubit. The operator $\hat{\varvec{R}}=2\left| {\phi }\right\rangle \left\langle {\phi }\right| -{\mathbb {I}}$ is the reflection with respect to the uniform superposition state, whereas the operator $\hat{\varvec{O}}_i={\mathbb {I}}-2\left| {x_i}\right\rangle \left\langle {x_i}\right|$ is built so as to flip the sign of the state $\left| {x_i}\right\rangle$ and leave all the other states unaltered. The Grover’s operator is applied until the amplitude of $\left| {x_i}\right\rangle$ approximates $a_i$ to the desired precision (see section “Details on the probability distribution encoding algorithm”). The state of the ancilla is not modified during the execution of the Grover’s algorithm.

The *i*-th step ends by ensuring that the amplitude of $\left| {x_i}\right\rangle$ is not modified anymore during the next steps. To this end, we ’tick’ this component by tying it to the state $\left| {0}\right\rangle _a$. This is obtained by applying the operator $\hat{\varvec{F}}_i=\left| {x_i}\right\rangle \left\langle {x_i}\right| \otimes \hat{\varvec{X}}+ \big ({\mathbb {I}}-\left| {x_i}\right\rangle \left\langle {x_i}\right| \big )\otimes \mathbbm {1}$, whose net effect is:

$$\begin{aligned} \hat{\varvec{F}}_i\left| {x_k}\right\rangle \left| {y}\right\rangle _a= {\left\{ \begin{array}{ll} \left| {x_k}\right\rangle \otimes \hat{\varvec{X}}\left| {y}\right\rangle _a , &{} \text{ if } k=i \\ \left| {x_k}\right\rangle \left| {y}\right\rangle _a, &{} \text{ otherwise } \end{array}\right. } \end{aligned}$$

Where $\hat{\varvec{X}}$ is the NOT-gate, with $1\le i \le J$, $y \in \{0,1\}$.

After the step *i*, the state of the system is:

$$\begin{aligned} \left| {\psi }\right\rangle= & {} \sum \limits _{j=1}^{i} a_j \left| {x_j}\right\rangle \otimes \left| {0}\right\rangle _a + b_{i} \left| {\beta _{i}}\right\rangle \left| {1}\right\rangle _a. \end{aligned}$$

The state $\left| {\beta _{i}}\right\rangle$ has in general non-zero overlap with all of the basis states, including those states, $\left| {x_1}\right\rangle$,$\cdots$,$\left| {x_{i-1}}\right\rangle$, whose amplitudes have been updated previously. This is due to the action of the reflection operator $\hat{\varvec{R}}$, which outputs a superposition of all the *J* basis states. However, this does not preclude us from extracting the value of the probability of the random variable correctly, thanks to the ancillary qubit. Indeed, at the end of the last step, the ancilla is first measured in the logical basis. If the outcome of the measurement is 0, then we can proceed measuring the rest of the QR to get one of the first $J-1$ values of the random variable with the assigned probability distribution. Otherwise, the output is set to $x_J$, since the probability of getting 1 from the measurement of the ancilla is $p_{x_{J}}$ due to the normalization condition ($p_{x_{J}}=a_{J}^2 = 1- \sum _{k=1}^{J-1} a_k^2$).

The procedure can be generalized to encode a random distribution for which *N* elements, with $N>J$, are divided into *J* sub-intervals. In this case, a probability is not assigned separately to every single element; but, rather, collectively to each of the *J* sub-intervals, while the elements belonging to the same sub-interval are assigned equal probabilities. This is useful to approximate a random distribution where the number of elements, *N*, is very large. In this case, the QR operates on an *N*-dimensional Hilbert space, and Grover’s operators are used to amplify more than one state at each of the $J-1$ steps. For example, in the simplest case, we can think of having a set of *J* Grover operators, each of which acts on *N*/*J* basis states. In the general case, though, each Grover’s operator could amplify a different, and a priori unknown, number of basis states. This case will be discussed in the following, where this quantum routine is exploited in the context of RL.

## Improving reinforcement learning

**Figure 1:**
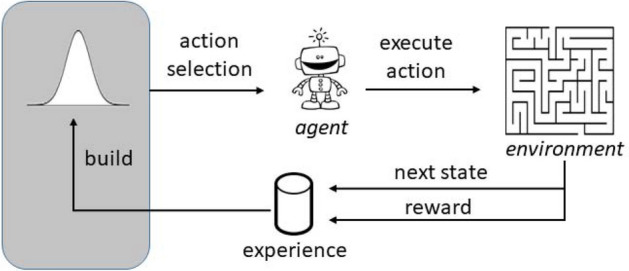
Sketch of the Q-learning cycle. An agent is schematically represented, performing a Q-learning cycle; namely, taking an action to solve a problem, getting back a reward and possibly changing its state and, finally, building a new probability distribution (by using the algorithm we are proposing), from which the next action is extracted.

We will now show how the algorithm introduced above can be exploited in the context of RL, and, specifically, in the Q-learning cycle. Figure 1 provides a sketch emphasising the part of the cycle that is involved in our algorithm. Our objective, in this context, is to update the action probabilities, as will be clarified in the rest of this section. An RL algorithm can be described in terms of an abstract *agent* interacting with an *environment*. The environment can be in one of the *states* *s* that belong to a given set *S* whereas the agent is allowed to perform *actions* picked from a set $A_s$, which, in general, depends on *s*. For each state $s \in S$, the agent chooses one of the allowed actions, according to a given policy. After the action is taken, the agent receives a *reward* *r* from the environment and its state changes to $s' \in S$. The reward is used by the agent to understand whether the action has been useful to approach the goal, and then to learn how to adapt and improve its behavior. Shortly, the higher the value of the reward, the better the choice of the action *a* for that particular state *s*. In principle, this behavior, or *policy*, should consist in a rule that determines the best action for any possible state. An RL algorithm aims at finding the optimal policy, which maximizes the overall reward, i.e., the sum of the rewards obtained after each action. However, if the rewards are not fully known in advance, the agent needs to act on the basis of an estimate of their values.

Among the various approaches designed to this end, the so called Q-Learning algorithm^4^ adopts the Temporal Difference (TD) method to update the *Q*(*s*, *a*) value, i.e., an estimation of how profitable is the choice of the action *a* when the agent is in the state *s*.

When choosing an action, at a given step of the algorithm, two key factors need to be taken into account: *explore* all the possible actions, and *exploit* the actions with the greatest values of *Q*(*s*, *a*). As it is common, we resort to a compromise between exploration and exploitation by choosing the new action through a random distribution, defined so that the probability of choosing the action *a* in the state *s* mirrors *Q*(*s*, *a*). For example, one could adopt a Boltzmann-like distribution $P(a|s)=e^{Q(s,a)/T}/Z$, where *Z* is a normalising factor, while the *T* parameter can vary during the learning process (with a large *T* in the beginning, in order to favor exploration, and lower *T* once some experience about the environment has been acquired, in order to exploit this knowledge and give more chances to actions with a higher reward).

A severe bottleneck in the performance of a TD training algorithm arises when the number of actions and/or states is large. For example, in the chess game, the number of states is $\sim 10^{120}$ and it is, in fact, impossible to deal with the consequent huge number of *Q*(*s*, *a*) values. A workaround is to use a function $Q^{*}_{\vec {\theta }}(s,a)$ that *approximates* the values of *Q*(*s*, *a*) obtained by the TD rule and whose properties depend upon a (small) set of free parameters $\vec {\theta }$ that are updated during the training. This approach showed its effectiveness in different *classical* approaches as, for example, in Deep Q-Learning^26–30^, where the $Q^{*}_{\vec {\theta }}(s,a)$ function is implemented by means of a neural network whose parameters are updated in accordance with the experience of the agent.

In the quantum scenario, this approach turns out to be even more effective. Indeed, it is possible to build a parameter-dependent quantum circuit that implements $Q^{*}_{\vec {\theta }}(s,a)$; an approach that has been adopted by recent studies on near-term quantum devices^31–35^. This circuit allows us to evaluate the function $Q^{*}_{\vec {\theta }}(s,a)$ in a complete *quantum parallel* fashion; i.e., in one shot for all the admissible actions in a given state. With this approach, it is possible to obtain a quantum advantage in the process of building the probability distribution for the actions, using the algorithm presented in section  “Preparing a quantum probability distribution”. To achieve a significant quantum speed-up, and reduce the number of required quantum resources, thus making our algorithm suitable for near term NISQ processing units, we do not assign a probability to every action; but, rather, we aggregate actions into classes (i.e., subsets) according to their probabilities, as explained in the following. Let us consider the minimum (*m*) and maximum (*M*) of the $Q^*_{\vec {\theta }}(s,a)$ values, and let us divide the interval [*m*, *M*] into *J* (non-overlapping, but not necessarily equal) sub-intervals $I_j$ with $1 \le j \le J$. For a given state *s*, we include the action $a\in A_{s}$ in the class $C_j$ if $Q^*_{\vec {\theta }}(s,a) \in I_j$ (so that, $A_s = \bigcup _j C_j$).

The probability of each sub-interval, then, will be determined by the sum of the $Q^*$-values of the corresponding actions, $\sum _{a \in C_j} Q^*_{\vec {\theta }}(s,a)$. All of the actions in $C_j$, will be then considered equally probable.

**Figure 2:**
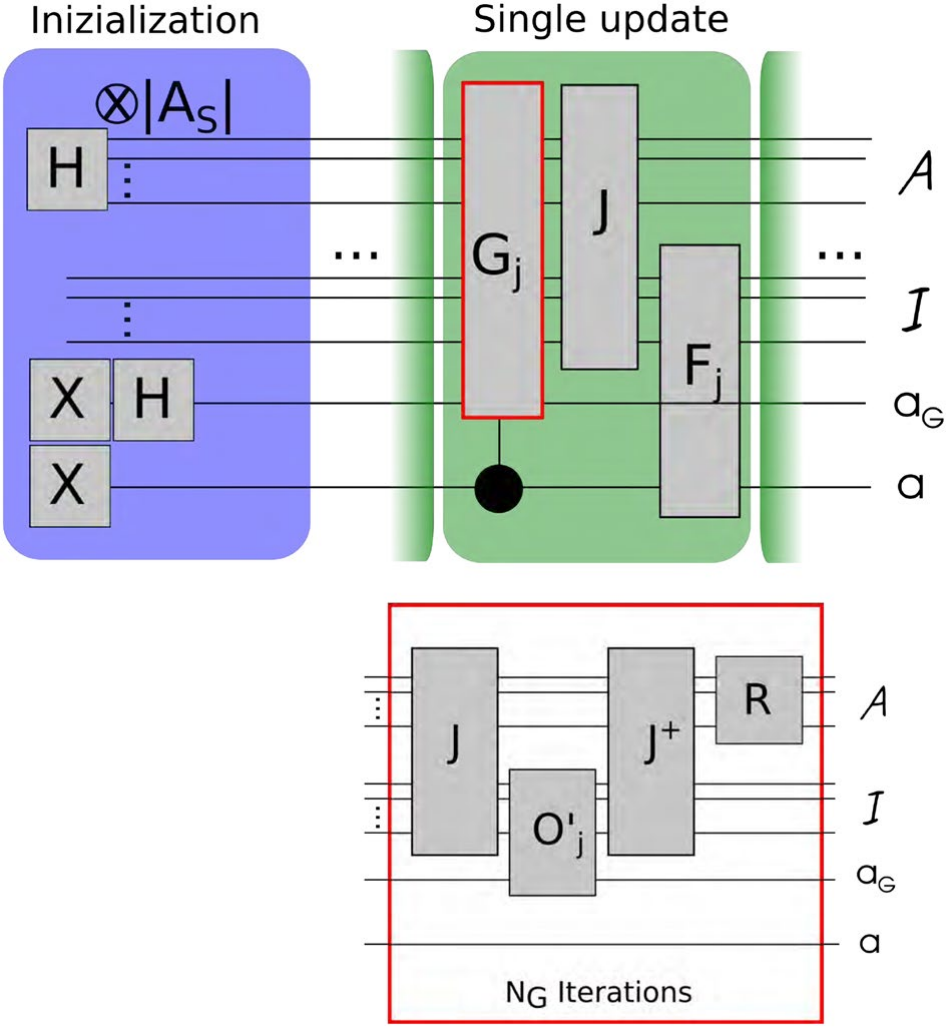
Quantum routine. Schematic representation of the quantum routine for the update of a classical probability distribution on the quantum register. After the initialization of the register the $J-1$ updates are performed to store the values of the probability distribution for each of the *J* classes for each state *s*. It shows also the single iteration of the modified Grover algorithm.

In this way, our algorithm requires only $J-1$ steps, each of which is devoted to amplify the actions belonging to one of the $J-1$ classes (while the *J*-th probability is obtained by normalization). Furthermore, we can also take advantage of the aggregation while encoding the probability distributions onto the QR: in this case, indeed, we can use predetermined Grover’s oracles, each devoted to amplify the logical states corresponding to the actions belonging to a given $C_j$.

For the offloading of the distribution-update routine onto a quantum processor as a part of the Q-learning procedure, we need two QRs: $\mathcal {A}$ and $\mathcal {I}$, which encode the actions and the sub-intervals, respectively. These registers need $\lceil log_2(\max _{s}|A_s|)\rceil$ and $\lceil log_2(J)\rceil$ qubits, respectively. Let us consider the class $C_j=\{a \in A_s : Q^{*}(s,a) \in I_j\}$. Our goal is to assign to each action $a \in C_j$ a probability $p_j$, based on the sub-interval *j*, using the routine presented in section “Preparing a quantum probability distribution”. The distribution building process starts by preparing the following uniform superposition:

$$\begin{aligned} \left| {\psi _{s}}\right\rangle =\frac{1}{\sqrt{|A_{s}|}}\sum _{a \in A_{s}}\left| {a}\right\rangle _{\mathcal {A}}\left| {0}\right\rangle _{\mathcal {I}}, \end{aligned}$$

where $\left| {0}\right\rangle _{\mathcal {I}}$ is the initial state of the register $\mathcal {I}$. In order to apply our algorithm, we need $J-1$ oracles $\hat{\varvec{O}}_j$, one for each given $I_j$. To obtain these oracles, it is first necessary to define an operator $\hat{\varvec{J}}$ that records the sub-interval $j_a$ to which the value $Q^{*}_{\vec {\theta }}(s,a)$ belongs. Its action creates correlations between the two registers by changing the initial state of the $\mathcal {I}$- register as follows:

$$\begin{aligned} \hat{\varvec{J}} \left| {a}\right\rangle _{\mathcal {A}}\left| {0}\right\rangle _{\mathcal {I}} = \left| {a}\right\rangle _{\mathcal {A}}\left| {j_a}\right\rangle _{\mathcal {I}}. \end{aligned}$$

To complete the construction of the oracles, we need to execute two unitaries: (i) the operator $\hat{\varvec{O}}'_{j_a}={\mathbb {I}}_\mathcal {A} \otimes ({\mathbb {I}}_\mathcal {I}-2\left| {j_a}\right\rangle \left\langle {j_a}\right| )$, which flips the phase of the state $\left| {j_a}\right\rangle$ of the $\mathcal {I}$-register; and, (ii) the operator $\hat{\varvec{J}}^{\dagger }$, which disentangles the two registers. The effective oracle operator entering the algorithm described in the previous section is then defined as $\hat{\varvec{O}}_{j}=\hat{\varvec{J}}^{\dagger }\hat{\varvec{O}}'_{j}\hat{\varvec{J}}$, its net effect being

$$\begin{aligned} \hat{\varvec{O}}_j \left| {a}\right\rangle _\mathcal {A}\left| {0}\right\rangle _\mathcal {I}={\left\{ \begin{array}{ll} -\left| {a}\right\rangle _\mathcal {A}\left| {0}\right\rangle _\mathcal {I}, &{} \text{ if } \text{ a } \in \,C_j \\ \left| {a}\right\rangle _\mathcal {A}\left| {0}\right\rangle _\mathcal {I}, &{} \text{ otherwise } \end{array}\right. } \end{aligned}$$

Eventually, we apply the reflection about average $\hat{\varvec{R}}$ on the register $\mathcal {A}$, thus completing an iteration of the Grover operator. Let us notice that whereas the operator $\hat{\varvec{R}}$ acts on the register of the actions, $\hat{\varvec{O}}'_{j}$ acts on the register of the classes and on the Grover ancilla. This is shown schematically in Fig. 2.

If the cardinality of each $C_j$ is not decided from the beginning, in order to evaluate the right number of Grover iterations to be executed, we need to compute it (see section “Optimal number of iterations of the Grover algorithm” for details). This number of actions can be obtained, for each $C_j$, by running the quantum counting algorithm associated with $\hat{\varvec{O}}_j$ (and before its action). It is then possible to apply the routine of section “Preparing a quantum probability distribution” in order to build the desired probability distribution. After the quantum state of the $\mathcal {A}$-register is obtained, the agent will choose the action measuring its state. Then, according to the outcome of the environment, it will update the $\vec {\theta }$ values classically, thus changing the behaviour of the operator $\hat{\varvec{J}}$.

We summarize the key steps of our quantum enhanced RL algorithm in the box below, where classical and quantum operations are denoted as (C) and (Q), respectively. A schematic picture of the amplitude distribution upload circuit is depicted in Fig. 2 where we show the needed resources as well as the main gates required.

**Table:** 

**Scheme of the hybrid algorithm**	
Initialize $\vec {\theta }$ and start from state *s* (C) Execute the cycle: $\bullet$ Build the sub-intervals, classes and the quantum circuits for the oracles $\hat{\varvec{O}}_i=\hat{\varvec{J}}^{\dagger }\hat{\varvec{O}}'_{i}\hat{\varvec{J}}$ (C) $\bullet$ Use quantum counting on $\hat{\varvec{O}}_i$ to compute the number of actions belonging to each sub-interval (Q) $\bullet$ Compute the number of Grover iterations for each class (C) $\bullet$ Build the probability distribution for the admissible actions in *s* (Q) $\bullet$ Measure, obtain an action and execute it (C) $\bullet$ Get the new state $s'$ and the reward *r* (C) $\bullet$ Update $\vec {\theta }$ (C)	

## Additional features of the algorithm

The quantum enhanced RL algorithm presented above resorts to quantum acceleration to remove bottlenecks of classical approaches. We provide, here, an assessment of the advantages obtained.

In the case of a finite and yet large number of actions $|A_{s}|\gg 1 \; \forall s$, one has that, classically, for a given state *s*, the number of calls to the function $Q^{*}_{\vec {\theta }}(s,a)$ increases asymptotically as ${\textbf {O}}(|A_{s}|)$. Conversely, with our quantum protocol the number of calls of $\hat{\varvec{J}}$, and therefore of $Q^{*}_{\vec {\theta }}(s,a)$, is asymptotically ${\textbf {O}}(\sqrt{|A_{s}|})$. Nonetheless, the larger the number of actions, the lower the bound on the error, which is of ${\textbf {O}}(1/\sqrt{|A_{s}|})$ (see section “Optimal number of iterations of the Grover algorithm” for details). Thus, as it is the case for all of the quantum algorithms based on Grover’s, we obtained a quadratic speed-up over the classical algorithm of updating a probability distribution.

Our strategy of discretizing the $Q^{*}_{\vec {\theta }}(s,a)$ values into bins affects also the reinforcement learning procedure, both from the point of view of the accuracy with which we are reproducing the desired probability distribution, and for the exploitation-exploration interplay. Let $\epsilon$ be the error introduced by the discretization with respect to the target probability distribution. In general, it may depend on features of the target distribution which are independent of the total number of actions and of bins. However, for a rough quantitative estimate, we can take it to scale proportionally to the size of the bins, obtaining the upper bound $\epsilon \sim \frac{M-m}{J}$, as a direct consequence of the rectangle method accuracy to approximate the integral. In fact, it is important that in the process of the definition of sub-intervals, the maximum (M) and the minimum (m) values of $Q^{*}_{\vec {\theta }}(s,a)$ are computable in advance by means of well-established quantum routines that do not increase the complexity of our procedure^36, 37^.

The discretization has also a direct impact on the number of calls needed to update the values of the probability distribution. Indeed, the larger the number of bins the more the calls to Grover oracle, which scale linearly with the number of bins (again, see section “Optimal number of iterations of the Grover algorithm” for details). The total computational time for the training is, in general, strictly problem dependent. It depends on the chosen cost function as well as other details of the RL. As such, the size of the bins, although not estimable *a priori*, is yet another parameter that can be used to lower the convergence time of the learning.

Finally, let us quantify the quantum resources needed in order to implement our algorithm. In the ideal noiseless case, as reported above, the qubits needed to implement our strategy can be organized into two registers: $\mathcal {A}$ and $\mathcal {I}$. The first one has to encode, in each case, all the actions of the reinforcement learning protocol and, as a consequence, it will require $log_2(\max _{s}|A_s|)\rceil$ qubits. The second one is, instead, devoted to encoding the *J* classes used to discretize the $Q^*$-values, needing $\lceil log_2(J)\rceil$ qubits. Moreover, ancillary qubits can be necessary to implement the oracle $\hat{\varvec{O}}_{j}$, but their number is strictly dependent on the specific problem. As a function of the number of actions, we can conclude that we have a logarithmic scaling of the number of required qubits.

In a more realistic scenario, in which decoherence affects the operations, one possibility to preserve the advantage is to resort to error correction algorithms. This unavoidably results in an increase in the amount of quantum resources. In our algorithm, the most sensitive part to noise is the amplification of the amplitudes via Grover’s algorithm on each interval. In Ref.^38^, an extensive analysis of the effect of noise on Grover search has been reported. The authors show that a [[7, 1]] Steane code^39^ is an effective strategy to correct errors in the Grover’s search algorithm. Allowing for a lower gain, the [[15, 7]] QBCH code^40^ can reduce the total amount of resource needed. As suggested in Ref.^38^, a hybrid approach can be considered, as a possible compromise between the two methods.

## Details on the probability distribution encoding algorithm

In this more technical section, we provide some details on the routine presented in section “Preparing a quantum probability distribution”, which are important for its actual implementation. Specifically, we address the problems of (i) how to compute the optimal number of iterations of the Grover algorithm in order to store a single instance of the probability distribution, (ii) how to compute quantities which are needed to link the update of the values of the probability distribution on different sub-intervals from one step to another, and, finally, (iii) evaluate its complexity.

### Optimal Number of iterations of the Grover algorithm

In order to compute the optimal number of iterations in a single step we exploit the results reported in Ref.^41^, where the Grover algorithm has been generalized to the case of an initial non-uniform distribution and define the following quantities :

$$\begin{aligned} {\bar{K}}^{(i)}(t)&=\frac{1}{r_{i}} \sum _{j=1}^{r_{i}} k_j^{(i)}(t)\\ {\bar{L}}^{(i)}(t)&=\frac{1}{N-r_{i}} \sum _{j=r_{i}+1}^{N} l_j^{(i)}(t) \end{aligned}$$

where t is the number of Grover iteration already performed, N is the dimension of the Hilbert space (in our context it is the total number of actions : $N=|A_s|$) , $\{k_j^{(i)}(t)\}$ are the coefficients of the $r_i$ basis states that will be amplified by the Grover iterations at the step *i* of the algorithm (in our application $r_i=|C_i|$), and $\{l_j^{(i)}(t)\}$ are the coefficients of all the other basis states, while ${\bar{K}}^{(i)}(t)$ and ${\bar{L}}^{(i)}(t)$ are their averages and we have labeled them with the step-counting variable *i*. Let’s assume now that only one basis state at a time is amplified by Grover iterations, namely $r_i=1$. Applying the results in Ref.^41^ to our case we obtain:

2 $$\begin{aligned} {\bar{K}}^{(i)}(t)= \,& {} k^{(i)}(0)\cos (wt)+{\bar{L}}^{(i)}(0)\sqrt{N-1}\sin (wt)\nonumber \\ {\bar{L}}^{(i)}(t)= \,& {} {\bar{L}}^{(i)}(0)\cos (wt)-k^{(i)}(0)\sqrt{\frac{1}{N-1}}\sin (wt), \end{aligned}$$

where $w=2\arcsin (\sqrt{1/N})$. With the first of these equations we can compute the number of steps $t_f^{(i)}$ needed to set the coefficient *k*(*t*) to the desired value with the wanted precision so as to bring the value of the probability distribution to $P(x_i)=|b_i\;k^{(i)}(t_f^{(i)})|^2$. Notice that we need the values of $k^{(i)}(0)$ and ${\bar{L}}^{(i)}(0)$ to perform this calculation, which values can be extracted from the form of the global state at the previous step and specifically from the last iteration of the Grover algorithm.

### Variation of quantum state within the Grover iterations

Let us consider the quantum state of the action-register plus the ancilla system at a given iteration *t* of the Grover algorithm at a given step *i*:

$$\begin{aligned} \left| {\psi (t)}\right\rangle =\sum _{k=1}^{i-1}a_k\left| {x_k}\right\rangle \left| {0}\right\rangle _a+b_i\left| {\beta _i(t)}\right\rangle \left| {1}\right\rangle _a \end{aligned}$$

where $b_i=(1-\sum _{k=1}^{j-1}a_k^2)^{1/2}$ and $t=0$ at the beginning of the Grover algorithm. Let us write the state $\left| {\beta _i(t)}\right\rangle$ in a form which highlights its decomposition into three sets of basis states:

3 $$\begin{aligned} \left| {\beta _i(t)}\right\rangle =k^{(i)}(t)\left| {x_i}\right\rangle + \sum _{k=1}^{i-1}l_k^{(i)}(t)\left| {x_k}\right\rangle +\sum _{k=i+1}^{N}\alpha ^{(i)}(t)\left| {x_k}\right\rangle , \end{aligned}$$

where we have made explicit the dependence of the coefficients of the decomposition on the step *i*, for this will be useful in the following. The $\left| {x_i}\right\rangle$ is the one we want to use to encode the value of the probability distribution at the current step *i* of our algorithm, the $\{x_j\}$ with $j\in [1,i-1]$ basis states that are generated by the reflection operation around the mean and whose amplitudes have been updated in the previous $i-1$ steps, and the $\{x_{j}\}$ with $j\in [i+1,N]$ basis state, all having the same amplitude $\alpha ^{(i)}(t)$ for all the operations performed up to this point did not change them.

Using this expression, it is possible to derive a recursive relation to compute $\alpha ^{(i)}(t)$ iteratively as a function of $\bar{L}^{(i)}(t)$ and $k^{(i)}(t)$. As we shall see below this will be useful in order to compute the initial $k^{(i+1)}(0)$,${\bar{L}}^{(i+1)}(0)$ and $\alpha ^{(i+1)}(0)$ for the next step. To find this recursive relation let us first apply the Grover operator onto $\left| {\beta _i(t)}\right\rangle \left| {1}\right\rangle _a$ and then project onto any of the states $\{x_{i+1}, x_{i+2}, \cdots , x_{N}\}$. Without loss of generality we choose $x_{i+1}$:

4 $$\begin{aligned}{} & {} \alpha ^{(i)}(t+1) = \left\langle {x_{i+1}}\right| \hat{\varvec{R}}\hat{\varvec{O}}_i\left| {\beta _i(t)}\right\rangle \nonumber \\{} & {} \quad = \left\langle {x_{i+1}}\right| \Bigl (2\left| {\phi }\right\rangle \left\langle {\phi }\right| -{\mathbb {I}}\Bigr )\nonumber \\{} & {} \qquad \times \left( -k^{(i)}(t)\left| {x_i}\right\rangle +\sum _{k=1}^{i-1}l_k^{(i)}\left| {x_k}\right\rangle + \sum _{k=i+1}^{N}\alpha ^{(i)}(t)\left| {x_k}\right\rangle \right) \nonumber \\{} & {} \quad = \left\langle {x_{i+1}}\right| \Bigl \{2\left( -\frac{1}{\sqrt{N}}k^{(i)}(t)+\frac{N-1}{\sqrt{N}}\bar{L}^{(i)}(t)\right) \left| {\phi }\right\rangle \nonumber \\{} & {} \qquad + k^{(i)}(t)\left| {x_i}\right\rangle -\sum _{k=1}^{i-1}l_k^{(i)}\left| {x_k}\right\rangle -\sum _{k=i+1}^{N}\alpha ^{(i)}(t)\left| {x_k}\right\rangle \Bigr \}\nonumber \\{} & {} \quad = \frac{2}{N} \bar{L}^{(i)}(t)(N-1)-\frac{2}{N}k^{(i)}(t)-\alpha ^{(i)}(t) \end{aligned}$$

Using Eqs. (2) and (4), as well as the initial values of $\alpha (0)$, *k*(0) and $\bar{L}(0)$, at the beginning of the Grover iterations, together with the number of iterations $t_f^{(i)}$, with a simple *classical* iterative procedure, we can compute the final values of $\alpha ^{(i)}(t_f^{(i)})$, $k^{(i)}(t_f^{(i)})$ and $\bar{L}^{(i)}(t_f)$. This concludes one step of the embedding algorithm.

### Linking two consecutive steps of the distribution-encoding algorithm

Let us see how to use the $\alpha ^{i}(t_f^{(i)})$, $k^{i}(t_f^{(i)})$ and $\bar{L}^{i)}(t_f^{(i)})$ computed at the end of step *i* to obtain the values $\alpha _{(i+1)}(0)$, $k^{(i+1)}(0)$ and $\bar{L}^{(i+1)}(0)$, which are needed in the following step $i+1$.

We first consider the global state at the end of the step *i* before applying the $\hat{\varvec{F}}_{i}$ operator to mark the state $\left| {x_i}\right\rangle$ whose amplitude has just been updated:

$$\begin{aligned} \left| {\psi (t_f^{(i)})}\right\rangle =\sum _{j=1}^{i-1}a_j\left| {x_j}\right\rangle \left| {0}\right\rangle _a+b_{i}\left| {\beta _{i}(t_f^{(i)})}\right\rangle \left| {1}\right\rangle _a \end{aligned}$$

where

5 $$\begin{aligned} \left| {\beta _{i}(t_f^{(i)})}\right\rangle =k^{(i)}(t_f^{(i)})\left| {x_{i}}\right\rangle +\sum _{k \ne {i}}l_k(t_f^{(i)})\left| {x_k}\right\rangle . \end{aligned}$$

After applying $\hat{\varvec{F}}_{i}$ we obtain the initial global state which will be the seed of the Grover iterations at the step $i+1$:

6 $$\begin{aligned} \left| {\psi (0)}\right\rangle =\sum _{k=1}^{i-1}a_k\left| {x_k}\right\rangle \left| {0}\right\rangle _a+a_{i}\left| {x_i}\right\rangle \left| {0}\right\rangle _a+b_{i+1}\left| {\beta _{i+1}(0)}\right\rangle \left| {1}\right\rangle _a, \end{aligned}$$

where

$$\begin{aligned} \left| {\beta _{i+1}(0)}\right\rangle =k^{(i+1)}(0)\left| {x_{i+1}}\right\rangle +\sum _{k \ne i+1}l_k^{(i)}(0)\left| {x_k}\right\rangle \end{aligned}$$

In this new state, the coefficient $b_{i+1}$ ensures that $\left| {\beta _{i+1}(0)}\right\rangle$ is unit. By looking at the coefficient of $\left| {x_{i}}\right\rangle$ in both (5) and (6), it is easy to see that $a_{i}=b_{i}k^{(i)}(t_f^{(i)})$. In the same way, we can compare the coefficients that in (6) appear with the state $\left| {1}\right\rangle _a$ of the ancilla with the corresponding components in (5):

$$\begin{aligned} b_{i+1}\left| {\beta _{i+1}(0)}\right\rangle \left| {1}\right\rangle _a= \,& {} b_{i}\Bigl (\left| {\beta _{i}(t_f^{(i)})}\right\rangle -k^{(i)}(t_f^{(i)})\left| {x_{i}}\right\rangle \Bigr )\left| {1}\right\rangle _a\\=\, & {} b_{i}\Bigl (\sum _{k \ne {i}}l_k^{(i)}(t_f^{(i)})\left| {x_k}\right\rangle \Bigr )\left| {1}\right\rangle _a \end{aligned}$$

It follows that:

$$\begin{aligned} \left| {\beta _{i+1}(0)}\right\rangle =\frac{b_i}{b_{i+1}}\sum _{k \ne i}l_k^{(i)}(t_f^{(i)})\left| {x_k}\right\rangle \end{aligned}$$

Finally we obtain that:

$$\begin{aligned} \alpha ^{(i+1)}(0)=k^{(i+1)}(0){} & {} =\frac{b_i}{b_{i+1}}\alpha ^{(i)}(t_f^{(i)})\\ \bar{L}^{(i+1)}(0){} & {} =\frac{\sum _{k \ne i+1}l_k^{(i+1)}(0)}{N-1}=\frac{b_i}{b_{i+1}}\frac{\sum _{k \ne i+1}l_k^{(i)}(t_f^{(i)})}{N-1} \\ {}{} & {} = \frac{b_i}{b_{i+1}}\left( \bar{L}^{(i)}(t_f^{(i)})-\frac{\alpha (t_f^{(i)})}{N-1}\right) \end{aligned}$$

It is also possible to generalize these results in the case in which the states to be updated are superposition of more than one basis states. Let us assume that we have $r_i>1$. In this case the general relations for $\bar{K^{(i)}}(t)$ and $\bar{L^{(i)}}(t)$ read :

$$\begin{aligned} {\bar{K}}^{(i)}(t)&=\bar{K}^{(i)}(0)\cos (wt)+{\bar{L}}^{(i)}(0)\sqrt{\frac{N-r_i}{r_i}}\sin (wt)\\ {\bar{L}}^{(i)}(t)&={\bar{L}}^{(i)}(0)\cos (wt)-\bar{K}^{(i)}(0)\sqrt{\frac{r_i}{N-r_i}}\sin (wt). \end{aligned}$$

with $w=2\arcsin (\sqrt{r_i/N})$. Since all the marked states have the same probability, we conclude that the probability of a single state is ${\bar{K}}^{(i)}(t)^2$. Moreover the expression for $\bar{L}^{(i+1)}(0)$ now is:

$$\begin{aligned} \bar{L}^{(i+1)}(0)=\frac{b_i}{b_{i+1}}\left( \frac{(N-r_i)\bar{L}^{(i)}(t_f^{(i)})-r_{i+1} \alpha ^{(i)}(t_f^{(i)})}{N-r_{i+1}}\right) , \end{aligned}$$

The other updating rules will be the same. It is important to underline that in this general case it is necessary to know in advance the number of states related to each oracle. As mentioned above, this can be set from the beginning or achieved by means of a quantum counting procedure.

### Complexity and precision

In order to compute the complexity of the algorithm, we start from the observation, derived in Ref.^41^, that the optimal number of Grover’s iterations for a given step i is upper bounded by :

$$\begin{aligned} N_I^{(i)}=\frac{\frac{\pi }{2}-\arctan \left( \frac{{\bar{K}}(0)}{{\bar{L}}(0)}\sqrt{\frac{r_i}{N-r_i}}\right) }{\arccos \left( 1-2\frac{r_i}{N}\right) }. \end{aligned}$$

Expanding $N_I^{(i)}$ to the leading-order in our working assumptions ($N>>1$), we obtain:

$$\begin{aligned} N_I^{(i)} \simeq -\frac{1}{2} \frac{{\bar{K}}(0)}{{\bar{L}}(0)}+\frac{\pi }{4}\sqrt{\frac{N}{r_i}}. \end{aligned}$$

From this expression, we can conclude that the initial conditions of the state can only reduce the optimal number of calls of Grover’s operators because we are dealing only with positive amplitudes, and we have ${\bar{K}}(0),{\bar{L}}(0) \ge 0$. It is worth to note that in our case we want to set the amplitude of each action not to the maximum value but to the value that is determined by a probability distribution, therefore the number of Grover’s iterations is typically much lower than the upper bound given above.

As explained in section “Improving reinforcement learning”, the number of times that the Grover procedure has to be executed is equal to the number of sub-intervals (J) chosen, and so the total complexity is:

$$\begin{aligned} {\textbf {O}}\left( J \sqrt{N}\right) ={\textbf {O}}\left( \sqrt{N}\right) , \end{aligned}$$

where we took into account that for a given state *s*, $N=|A_{s}|$, the complexity is equal to ${\textbf {O}}\left( \sqrt{|A_{s}|}\right)$.

A useful quantity to compute in a practical implementation of the algorithm is the precision $\Delta P$. It is the variation of the probability associated to an action between two consecutive iterations of the amplitude amplification (from *t* to $t+1$). This can be used as a criterion to stop the iterations. It can be quantified as follows:

7 $$\begin{aligned} \Delta P=\, & {} |\left\langle {x_i}\right| \left\langle {1}\right| _a\left| {\psi (t+1)}\right\rangle |^2-|\left\langle {x_i}\right| \left\langle {1}\right| _a\left| {\psi (t)}\right\rangle |^2\nonumber \\= \,& {} b_i^2\Bigl (\bar{K}(t+1)^2-{\bar{K}}(t)^2\Bigl )\nonumber \\= \,& {} b_i^2\Bigl ({\bar{K}}(0)^2[\cos (w(t+1))^2 -\cos (wt)^2]\nonumber \\{} & {} + {\bar{L}}(0)^2\bigl (\frac{N-r_i}{r_i}\bigl )[\sin (w(t+1))^2-\sin (wt)^2]\nonumber \\{} & {} +{\bar{K}}(0) {\bar{L}}(0) \sqrt{\frac{N-r_i}{r_i}} [\sin (2w(t+1))-\sin (2wt)]\Bigl ). \end{aligned}$$

Recalling that we assumed $|A_{s}|>>1$ and considering the upper bound case in which $t \sim \sqrt{|A_{s}|}$ and $r_1<<N$, we can expand $\Delta P$ to the leading-order in $1/|A_{s}|$, we have:

$$\begin{aligned} \Delta P&\sim b_i^2\Bigl ({\bar{K}}(0)^2\frac{1}{\sqrt{|A_{s}|}}+{\bar{L}}(0)^2|A_{ s}|\frac{1}{\sqrt{|A_{s}|}}\\&\quad +{\bar{K}}(0) {\bar{L}}(0) \sqrt{|A_{s}|}\Bigl ) \end{aligned}$$

Interestingly, the precision is bounded from below and the bound is obtained for $b_i=1$, $K(0) \sim |A_{s}|^{-1/2}$ and $\bar{L}(0) \sim |A_{s}|^{-1/2}$:

$$\begin{aligned} \Delta P \sim \frac{1}{\sqrt{|A_{s}|}}. \end{aligned}$$

The quantity $\Delta P$ can also be seen as the minimum error on the probability update, no matter how many iterations we perform.

## Conclusions

In our work, we presented a routine, based on the Grover’s algorithm, to encode a probability distribution onto a quantum register with a quadratic speed-up improvement. This quantum routine can find several useful applications in the context of hybrid classical-quantum workflows. In this spirit, we have shown how to exploit it for the training of the Q-learning strategy. We have shown that this gives rise to a quadratic quantum speed up of the RL algorithm, obtained by the inclusion of our quantum subroutine in the stage of action selection of the RL workflow for a large but finite number of actions. This effectively enables achieving a trade off between exploration and exploitation, thanks to the intrinsic randomness embodied by the extraction from a QR of the action to be performed and, also, to the possibility of dynamically changing the relationship between the action and their values (and, thus, their relative probabilities). In the classical case, the trade off between exploitation and exploration needs to be implemented as an extra control parameter, typically via a random variable and a user-defined threshold that manages the rate of acceptance of non-optimal stat-action pairs.

Finally, we stress once again that, with our procedure, we can use Grover’s oracles, which are given once and for all if i) the minimum and maximum range of action values, and ii) the number of intervals in which this range is divided are specified in advance.

## Data Availability

All data generated or analysed during this study are included in this published article.
